# The neutrophil-to-Lymphocyte ratio is associated with clinical symptoms in first-episode medication-naïve patients with schizophrenia

**DOI:** 10.1038/s41537-024-00437-5

**Published:** 2024-02-03

**Authors:** Xuan Wang, Xiaofang Chen, Xiaoni Guan, Zezhi Li

**Affiliations:** 1 Hebei Province Veterans Hospital, Baoding, China; 2 Peking University HuiLongGuan Clinical Medical School, Beijing HuiLongGuan Hospital, Beijing, China; 3 Department of Nutritional and Metabolic Psychiatry, The Affiliated Brain Hospital of Guangzhou Medical University, Guangzhou, China; 4 Guangdong Engineering Technology Research Center for Translational Medicine of Mental Disorders, Guangzhou, China; 5 Key Laboratory of Neurogenetics and Channelopathies of Guangdong Province and the Ministry of Education of China, Guangzhou Medical University, Guangzhou, China

**Keywords:** Schizophrenia, Biomarkers

## Abstract

Innate immunity has been shown to be associated with schizophrenia (Sch). This study explored the relationship between symptoms and neutrophil-to-lymphocyte ratio (NLR) (a marker of innate immunity) in patients with Sch. Ninety-seven first-episode medication-naïve (FEMN) patients with Sch and 65 healthy controls were recruited in this study. We measured the complete blood count and assessed the clinical symptoms using the PANSS scales. We found higher NEU counts and NLR in patients with Sch compared with control subjects. Male patients showed a higher NEU count than female patients. In addition, FEMN patients with higher NLR and NEU values showed higher PANSS-p, PANSS-g, and PANSS-total scores (all *p* < 0.05). Regression analysis revealed that NLR was a predictor for PANSS total scores in patients with Sch. Higher NLR value was observed in patients with Sch and the significant associations between NLR and psychotic symptoms indicate that an imbalance in inflammation and innate immune system may be involved in the pathophysiology of Sch.

## Introduction

Schizophrenia (Sch) is a complex psychiatric disorder manifesting as psychotic symptoms, negative symptoms, and cognitive impairments. The pathophysiological mechanism underlying Sch remains unknown and is partially due to its heterogeneous genetic, neurobiological, and phenotypic profile. Current antipsychotic medications have been reported to be beneficial in only about 30% of patients with Sch^1–3^. The vulnerability-stress-inflammation model has received increasing interest and suggests that prenatal exposure to influenza virus, toxoplasmosis, herpes viruses or other viruses is considered to be an environmental neurodevelopmental trigger for psychotic symptoms and that infection during childhood and adolescence may trigger episodes or relapses of Sch in genetically susceptible people^4–11^.

Inflammation is a natural and essential response of the body to foreign pathogens to activate immune cells and molecular mediators, such as cytokines and complement proteins^12,13^. Although peripheral cytokines can cross the blood-brain barrier and invade the central nervous system^14^, the brain may be attacked by inflammation through the activation of microglia, the innate immune cells in the brain^15,16^. It is now generally accepted that microglia are primed during early brain development and then switch to a pro-inflammatory state in response to stress during critical periods of development, which in turn leads to neurotransmission abnormalities, synaptic pruning, and structural damage to neurons^17^. The roots of immune abnormality as a potential predictive biomarker for Sch lie in the observed association between microglia-mediated inflammatory processes and the development of psychosis. There is accumulating evidence for the presence of abnormal immune system markers, including chronic immunity and inflammatory biomarkers in the pathophysiology of Sch in postmortem studies and studies examining cytokines in cerebrospinal fluid of patients^18–21^. In particular, the previously identified genetic locus of vulnerability to Sch includes gene-coding immune-related components^22,23^. Moreover, anti-inflammatory agents in combination with antipsychotics have shown better efficacy in improving clinical symptoms as compared with treatment with antipsychotic drugs alone^24^. Furthermore, recent studies have shown that first-degree relatives of patients with Sch have altered levels of inflammatory markers, increasing the likelihood of inflammation-related abnormalities as a promising endophenotype for Sch.

Peripheral white blood cells are important components of the immune system. Complete blood cell count is a widely used non-specific marker of inflammatory state^25^. Altered complete blood cell counts have been reported in patients with Sch, especially lymphocyte (LYM) and neutrophils (NEU), compared to healthy controls. NEU reflects non-specific inflammation and plays a role in the body’s innate immunity, resulting in a rapid response to infection^26^. LYM indicates specific immunity and is important for the body’s adaptive immunity^27,28^. Increased NEU counts have been reported in patients with first-episode Sch and were associated with PANSS total scores^29,30^. In addition, a recent meta-analysis also revealed an increase in LYM counts in first-episode patients^31^. In addition to these two indicators, the neutrophil-to-lymphocyte ratio (NLR) is also a measure of the overall inflammatory reaction and reflects the balance between two opposing but complementary pathways^32^. Thus, NLR has recently been shown to be a novel marker for systemic inflammation derived from routine blood cell counts. In addition, increased NLR values were found to be correlated with an elevation in cytokines and CRP and have been widely used as a proxy of systemic inflammation in recent studies^33–35^. A recent meta-analysis of 804 patients with Sch and 671 controls reported elevated NLR in first-episode patients with Sch^36^. Moreover, the authors found that antipsychotic medication affected patients’ NLR^36^. However, the findings of the relationship between NLR and clinical symptoms in patients with first-episode Sch were controversial^37^.

Considering that NLR is a relatively new biomarker in Sch and few studies have investigated the potential involvement in the disease pathophysiology, this study was designed to assess whether NLR was associated with the clinical symptoms evaluated by the PANSS in first patients with Sch after controlling for the confounding factors. This study would answer the following questions: 1) Were there differences in NEU, LYM, and NLR, between first-episode medication-naive (FEMN) patients and healthy controls? and 2) Is there an association between NEU, LYM and NLR and clinical symptoms in FEMN patients with Sch?

## Methods

### Participants

This study was designed as a multi-center and cross-sectional observational study of the FEMN patients. We conducted this study at Hebei Province Veterans Hospital and Beijing Huilongguan Hospital. The diagnostic eligibility of participants was determined using the SCID^38^. The research protocol was approved by the Institutional Review Board of Beijing Huilongguan Hospital and written informed consent was obtained from the participants or their parent/legal guardian/next of kin to participate in the study.

The inclusion criteria were: 1) Han ethnicity; 2) age between 16 and 45 years; 3) duration of illness≤ 5 years; and 4) no previous treatment or cumulative use of oral antipsychotic medication ≤ 14 days. Exclusion criteria included: 1) substance abuse/dependence; 2) severe somatic diseases; 3) self-injury, intrusive agitation, destructive behaviour, or suicide; 4) use of oral anti-immune agents; and 5) with ongoing infections, allergies or history of autoimmune disorders. Finally, a total of 97 FEMN patients with Sch were recruited in this study.

A total of 65 control subjects were recruited in our study. Healthy controls were excluded if they were diagnosed with major Axis I disorder or had a family history of psychiatric disorders. Medical histories and physical examinations were obtained from each participant.

### Clinical assessment

The clinical symptoms were assessed using the Positive and Negative Syndrome Scale (PANSS)^39^. Experienced interviewers assessed the symptoms of patients. They received comprehensive training and the intra-class correlation coefficients (ICCs) for the total score exceeded 0.8.

### Determination of blood cell counts

Blood samples were extracted from each patient between 7:00 and 8:00 am. Complete blood count was conducted using the SYSMEX XN-3000 assembly line (Sysmex Corporation, Japan). The measure was carried out in strict accordance with the operating manual and was quality-controlled. LYM and NEU count data for each patient was obtained from the laboratory electronic system at the hospital.

### Statistical analysis

Sample size power was calculated based on expected differences in NLR values. The sample size in our study was considered to achieve significance with a large effect size (ES) (d = 0.50), a power of 80%, and α = 0.05. Demographic data, baseline PANSS scores, LYM and NEU counts were compared in patients and healthy controls. The normality of the distribution of LYM and NEU counts and NLR values was assessed using the Shapiro-Wilk test. The normally distributed continuous variable was reported by mean ± standard deviation (SD) and was compared using the univariate analysis of variance. Non-normally distributed continuous variables were expressed median and interquartile ranges and were compared using the nonparametric Wilcoxon tests. The categorical variable was reported as absolute numbers and percentages, and was compared using the *X*^2^ test.

Since NLR was not normally distributed in patients and controls, Spearman’s nonparametric bivariate correlations analyses were conducted to analyze the associations of LYM and NEU counts and NLR values with clinical symptoms and demographic and clinical data in patients. Further regression analysis was performed to investigate the potential relevant variables for the clinical symptoms and the following variables were included in the models: demographic data and blood cell count.

Bonferroni correction was conducted to adjust for multiple comparisons. All analyses were performed using the SPSS version 20.0. *P* < 0.05 was set as the threshold for significance.

## Results

### Comparisons between patients and healthy controls

The values of NEU and LYM, NRI, sex, age, education, age of onset and BMI of the patients and controls are shown in Table 1. No significant differences were observed in age, sex, age of onset and BMI between the two groups.

**Table 1:** Demographic data and clinical characteristics between patients and controls at baseline by using univariate analysis of variance, nonparametric analysis and chi-square.

	Sch patients	Healthy controls	
	(*n* = 97)	(*n* = 65)	F or Z or *X*^2^ (*p*)
Age (ys)	24.1 ± 6.9	24.8 ± 6.1	0.4 (0.51)
Education (ys)	8.1 ± 2.8	11.5 ± 3.4	46.5 (<0.001)
Sex (Male, %)	55 (56.7%)	42 (64.6%)	1.0 (0.31)
BMI (kg/m^2^)	21.3 ± 3.4	21.4 ± 3.2	0.2 (0.68)
Onset age (ys)	23.4 ± 6.9		
NLR, median (IQR)	2.1 (1.6, 3.3)	1.6 (1.3, 2.2)	3.0 (0.003)
LYM count (10^9^/L)	2.0 ± 0.8	2.3 ± 0.7	3.3 (0.07)
NEU count (10^9^/L)	4.4 ± 1.7	3.9 ± 1.4	4.1 (0.045)

Note: *BMI* Body mass index, *ys* years, *LYM* lymphocytes, *NEU* neutrophils, *NLR* neutrophil/lymphocyte ratio.

The median NLR values for the patient group were 2.1 (IQR: 1.6, 3.3, 95% CI: 2.2–2.8) and the median NLR values for the control group were 1.6 (IQR: 1.3, 2.2, 95% CI: 1.7–2.1). The mean NEU counts were 4.4 (SD: 1.7, 95% CI: 1.9–2.2) in the patient group and 3.9 (SD: 1.4, 95% CI: 3.6–4.3) in the control group. NLR values and NEU counts were higher in FEMN patients with Sch compared with controls (for NLR: Z = 3.0, *p* = 0.003; for NEU counts: F = 4.1, *p* = 0.045). After Bonferroni corrections, the difference in NLR remained significant (Bonferroni corrected *p* = 0.009). There was no significant difference in LYM counts between the two groups.

Correlation analysis revealed no significant associations between age, age of onset, BMI, and counts of NEU and LYM or NLR values in patients with Sch (all *p* > 0.05).

### Association between NLR and clinical symptoms

As shown in Table 2, Spearman nonparametric bivariate correlation analyses revealed significant positive associations between NLR and negative symptoms (r = 0.25, *p* = 0.01), general psychopathology (r = 0.34, *p* = 0.001), and total scores (r = 0.33, *p* = 0.001) (Table 2). Additionally, we found that NEU counts were also positively associated with negative symptoms (r = 0.27, *p* = 0.008), general psychopathology (r = 0.27, *p* = 0.008) and total scores (r = 0.36, *p* < 0.001). After Bonferroni corrections, correlations between NLR or NEU counts and PANSS total scores (NLR: Bonferroni corrected *p* = 0.012 and NEU: Bonferroni corrected *p* = 0.0036), as well as between NLR and general psychopathology remained significant (Bonferroni corrected *p* = 0.012).

**Table 2:** Correlations between NLR and clinical symptoms using Spearman nonparametric bivariate correlations analyses.

	P subscale		N subscale		G subscale		Total score	
	r	*p*	r	*p*	r	*p*	r	*p*
NLR	0.16	0.12	0.25	0.01	0.34	0.001*	0.33	0.001*
LYM	0.02	0.82	-0.06	0.56	-0.15	0.14	-0.05	0.61
NEU	0.16	0.12	0.27	**0.008**	0.27	**0.008**	0.36	<0.001*

Note: *After Bonferroni correction, *p* < 0.05. Bold *p* values mean significant.

**Fig. 1:**
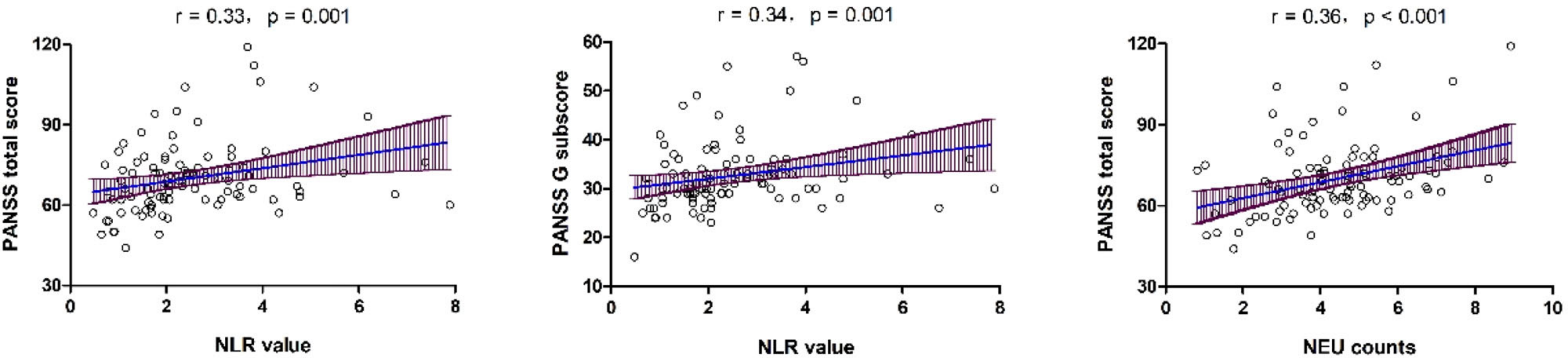
There were significant associations between NEU counts or NLR values and clinical symptoms assessed by PANSS in first-episode and drug-naïve patients with schizophrenia.

Because of the close relationship between age and age of onset (r = 0.98), and to avoid including completely collinear covariates in the multivariate analysis, we chose age for the regression analysis. After covarying for confounding factors such as age, sex, and years of education, the regression analysis revealed that NLR was a predictor for PANSS total scores in FEMN patients with Sch (β = 2.929, t = 3.489, *p* = 0.001; 95% CI: 1.261–4.598) (*R*^2^ = 0.247) (Table 3 and Fig. 1). In addition, NEU counts were also an independent predictor for clinical symptoms in patients (β = 2.894, t = 4.007, *p* < 0.001; 95% CI: 1.459–4.329) (*R*^2^ = 0.274) (Table 3 and Fig. 1).

**Table 3:** Regression analysis of the associations between NLR and NEU counts and clinical symptoms.

	Regression analysis				
*NLR values*	B (95% CI)	SE	β	t	p
Sex	0.566(−4.380 to 5.512)	2.489	0.021	0.227	0.821
Age	−0.581(−0.942 to −0.219)	0.182	−0.294	−3.191	0.002
Education years	−1.449(−2.315 to −0.582)	0.436	−0.305	−3.321	0.001
NLR values	2.929(1.261 to 4.598)	0.840	0.328	3.489	0.001
***NEU counts***					
Sex	1.210(−3.691 to 6.111)	2.467	0.046	0.490	0.625
Age	−0.396(−0.755 to −0.036)	0.181	−0.201	−2.187	0.031
Education years	−1.281(−2.132 to −0.429)	0.429	−0.270	−2.988	0.004
NEU counts	2.894(1.459 to 4.329)	0.722	0.377	4.007	<0.001

Note: *NEU* neutrophils, *NLR* neutrophil/lymphocyte ratio, *B* unstandardized regression coefficient, *β* standardized regression coefficient, *t* t statistic, *SE* standard error.

For NLR: ANOVA, F = 7.370; *p* < 0.001; adjusted *R*^2^ = 0.247.

For NEU counts: ANOVA, F = 8.507; *p* < 0.001; adjusted *R*^2^ = 0.274.

## Discussion

We revealed significant increases in NEU counts and NLR values in FEMN patients relative to healthy controls. NLR and NEU counts correlated with clinical symptoms assessed by the PANSS scale after controlling for sex, age, BMI, and age of onset.

Our finding of higher NEU counts in patients compared to healthy controls is consistent with previous studies in patients with Sch^25,29,30^, suggesting the involvement of increased NEU counts in the pathogenesis of Sch. The immune hypothesis of Sch has become increasingly prominent in the study of its etiology^40–42^, including maternal viral infection and immune activation, as well as variants of immune genes that contributed to Sch risk. NEUs are the most abundant leukocytes in peripheral blood cells and are considered to be the first line of defense of the innate immune system^26^. In addition to its phagocytosis, NEU secretes more than 70 different pro-inflammatory compounds, including cytokines, chemokines, and growth factors, which provoke inflammatory responses and oxidative stress, resulting in a rapid response to infection^43,44^. It should be noted that there was no difference in LYM counts between patients and controls in our study, indicating that adaptive immunity was not involved in the onset of this disease. Our findings provide further evidence for the role of innate immune cells as modulators of acute disease severity.

Furthermore, we found that NLR was increased in patients with Sch relative to healthy controls and that increased NLR was positively correlated with psychotic illness severity score. The NLR, calculated by dividing the absolute NEU count by the absolute LYM count, is an important inflammatory marker that is easily measured, repeatable, and inexpensive from routine blood testing^44^. It is a more reliable indicator of the intensity of stress and systemic inflammation. In line with our findings, two recent meta-analyses have shown evidence of higher NLR values in individuals with non-affective psychosis^36,45^. In addition, NEU counts have been reported to be influenced by age, sex, nicotine, and BMI^46^. Notably, in the present study, patients were well-matched and specifically controlled for several confounders, such as sex, age, years of education, and BMI. We also assessed the effects of sex, years of education, age, and BMI on NLR in patients and found no associations. Therefore, the results of increased NLR found in our study were not correlated with these confounding factors, but maybe with Sch itself.

Consistent with previous studies, we found that NEU counts and NLR were associated with clinical symptoms^30,47,48^, suggesting that NEU may be involved in the pathophysiology of Sch. The potential mechanisms that may be behind the increased NLR values in Sch, primarily cytokine system disorders and their impact on the activation of the immune system. A growing and compelling body of evidence demonstrates that patients suffering from Sch have the baseline changes in proinflammatory and anti-inflammatory cytokine levels throughout the disease and immunologic dysfunctions as a key element in its pathophysiology^20,49,50^. However, inconsistent findings from previous studies regarding the associations between NLR values and clinical symptom severity scores have limited the conclusions. For example, two studies reported no significant association with the severity of clinical symptoms assessed by the Brief Psychiatric Rating Scale, Clinical Global Impression, PANSS, or disease duration^51,52^. Possible explanations for these controversial findings may be that the patients recruited were at different stages of Sch or the type and dose of antipsychotic medication or treatment duration. Previous studies have shown the potential impact of antipsychotics on NLR values^53^. The patients in our study were FEMN patients, while other studies recruited patients treated with antipsychotics.

The present study benefited from a sample of drug-naïve Sch patients, given the inherent challenges of recruiting FEMN patients. In particular, patients and controls were well-matched in most demographic characteristics. However, several limitations should also be noted in this study. Firstly, given the cross-sectional design we used and we only examined our patients once, this study cannot draw any causal relationship regarding the associations of NLR and clinical symptoms in Sch. A further longitudinal study and comparisons between naïve and long-term medicated states could likely better demonstrate the relationship between them. Secondly, although we controlled for some confounding factors, some other factors associated with immune activation and inflammation were neglected. These factors are particularly important for first-episode patients with Sch, as many of these factors were exacerbated when patients experience their first episodes, such as depression, anxiety, stress, and related sleep abnormalities. However, these data were not collected and would need to be investigated in future short- or long-term longitudinal studies. Thirdly, we only measured the NEU and LYM counts in white blood cells, and we did not measure other immune cells or related cytokines, chemokines, and components. As previous studies have shown that NEU and LYM were significantly correlated with cytokines, chemokines, and growth factors, the interrelationships between white blood cells and cytokines need further investigation in further studies. Forth, the detailed duration of the disease of recruited patients was not collected in our study. However, all recruited participants were first-episode patients and their duration of the disease was <= 5 years to meet the inclusion/exclusion criteria. Fifth, NEU counts can be influenced by nicotine consumption, and nicotine addiction is very prevalent in patients with Sch. However, we did not report the smoking rates in our study, nor did we control for this variable in the comparisons.

## Conclusion

The present study demonstrates that increased NEU counts result in the high NLR value in patients with Sch. Our findings provide further evidence for the existence of increased NLR in Sch. More importantly, our results revealed that increased NLR was associated with psychotic symptoms at the first episode of symptoms. Notably, our findings may not be generalized to other patients with different ethnic and chronic Sch receiving antipsychotic medication.

## Data Availability

The data that support the findings of this study are available on request from the corresponding author. The data are not publicly available due to privacy or ethical restrictions.
